# The Synergistic Effect of Topographic Factors and Vegetation Indices on the Underground Coal Mine Utilizing Unmanned Aerial Vehicle Remote Sensing

**DOI:** 10.3390/ijerph20043759

**Published:** 2023-02-20

**Authors:** Quansheng Li, Feiyue Li, Junting Guo, Li Guo, Shanshan Wang, Yaping Zhang, Mengyuan Li, Chengye Zhang

**Affiliations:** 1 State Key Laboratory of Water Resource Protection and Utilization in Coal Mining, CHN Energy Shendong Coal Group Co., Ltd., Ordos 017209, China; 2 Department of Ecological Restoration, National Institute of Clean-and-Low-Carbon Energy, Beijing 102211, China; 3 College of Geoscience and Surveying Engineering, China University of Mining and Technology, Beijing 100083, China; 4 Geological Hazard Investigation and Monitoring Center, China Aero Geophysical Survey and Remote Sensing Center for Natural Resources, Beijing 100083, China

**Keywords:** UAV remote sensing, underground coal mine, slope, aspect, normalized difference vegetation index

## Abstract

Understanding the synergistic effect between topography and vegetation in the underground coal mine is of great significance for the ecological restoration and sustainable development of mining areas. This paper took advantage of unmanned aerial vehicle (UAV) remote sensing to obtain high-precision topographic factors (i.e., digital elevation model (DEM), slope, and aspect) in the Shangwan Coal Mine. Then, a normalized difference vegetation index (NDVI) was calculated utilizing Landsat images from 2017 to 2021, and the NDVI with the same spatial resolution as the slope and aspect was acquired by down-sampling. Finally, the synergistic effect of topography and vegetation in the underground mining area was revealed by dividing the topography obtained using high-precision data into 21 types. The results show that: (1) the vegetation cover was dominated by “slightly low-VC”, “medium-VC”, and “slightly high-VC” in the study area, and there was a positive correlation between the slope and NDVI when the slope was more than 5°. (2) When the slope was slight, the aspect had less influence on the vegetation growth. When the slope was larger, the influence of the aspect increased in the study area. (3) “Rapidly steep–semi-sunny slope” was the most suitable combination for the vegetation growth in the study area. This paper revealed the relationship between the topography and vegetation. In addition, it provided a scientific and effective foundation for decision-making of ecological restoration in the underground coal mine.

## 1. Introduction

Underground mining is a downward mining process utilizing a system of wellbore and underground laneways [1]. Compared with the direct destruction of land and vegetation by surface mining, underground mining has less damage to the surface vegetation [2]. However, this mining way easily leads to surface subsidence and deformation and to changes in the surface slope and aspect, which indirectly impact the vegetation growth on mining areas [3]. Slope and aspect are typical topographic factors [4]. Changes in slope cause fluctuations in soil water holding capacity, resulting in an impact on water availability for vegetation growth processes. The aspect affects the light availability for vegetation growth by changing the light intensity [5,6]. Therefore, slope and aspect are the key factors affecting the environment of the vegetation growth [7]. Exploring the relationship between the topography and vegetation can provide guidance for the decision-making of vegetation restoration in an underground coal mine.

Vegetation is the most important for ecological restoration in mining areas. The spectral reflection curve of vegetation has strong absorption characteristics in the red band and high reflection characteristics in the near-infrared band, resulting in a large difference between the reflection in the red band and near-infrared band. Therefore, scholars have proposed various vegetation indices, such as the ratio vegetation index (RVI) [8], normalized difference vegetation index (NDVI) [9], enhanced vegetation index (EVI) [10], etc. The existing research usually calculated one or several vegetation indices, and then mathematical statistical methods were used to conduct an analysis of vegetation change or relationship between vegetation and other factors from a temporal and spatial perspective. For example, Bao et al. used the NDVI, EVI, soil-adjusted vegetation index (SAVI), and transformed soil-adjusted vegetation index (TSAVI) to monitor the restoration of vegetation in mining areas. These works provided insight into vegetation on monitoring the restoration effect [11,12]. Furthermore, Ma et al. and Yang et al. analyzed the variation trend of interannual vegetation cover in a mining area from the perspective of time [13,14]. The spatial pattern change of vegetation cover in a mining area was analyzed from the perspective of space by Mi et al. and Dlamini et al. [15,16]. In these publications, selecting the NDVI as the vegetation indicator is the most common in mining areas [17,18,19]. 

The existing literature demonstrated that temporal and spatial variation of the NDVI is very sensitive to elevation and slope [20]. On natural sites, different topographic factors have different impacts on the NDVI [21]. For example, Zhu et al. discovered that the NDVI was shown to be proportional to slope in the Qinling Mountains, and the aspect had no significant impact on the NDVI [22]. Mokarram et al. found that there were positive and significant correlations between the NDVI and landform in the southwest of Fars province, Iran [23]. Furthermore, in the existing literature, the topographic factors (i.e., slope and aspect) were mainly obtained by a digital elevation model (DEM) [24]. There are two main approaches to obtain DEMs: (1) The DEM is built through field investigation [25]. First, data collection and contour line drawing are carried out, and then the contour line is used for data interpolation to generate the regular grid, to represent ground elevation. (2) The DEM is directly downloaded from the Geospatial Data Cloud (http://www.gscloud.cn, accessed on 24 December 2022) or other ways [26], which is widely used in existing studies, and its research scope is large (not limited to a mine). However, the DEM obtained by the first approach cannot accurately represent the true terrain structure, and this approach has complex data processing and a cumbersome workload. The data acquired by the second approach are easily impacted by the climate and environment, leading to many missing pixels indeterminately [27]. Furthermore, the spatial resolution of the DEM obtained by the second approach is usually at a low level, typically 30 m, which cannot meet the requirements of the detailed and small-scale analysis in underground mining areas.

Unmanned aerial vehicle (UAV) remote sensing has been becoming an effective modern monitoring tool equipped with monitoring devices, with a large photographic range and more accurate captured data [28,29]. Therefore, UAV remote sensing is characterized by high operational efficiency, low maintenance costs, high accuracy, and simple operation. UAVs can perform high-speed photography of the surface affected by underground mining, so that surficial high-precision topographic data can be obtained [30]. It provides a technical and equipment guarantee for exploring the relationship between topographic factors and vegetation on underground mining areas.

In this paper, high-precision DEM data were obtained by the UAV, and the precious slope and aspect of the study area were calculated by the DEM. It has been verified that the DEM data generated by the UAV were consistent with the actual elevation, and it was able to accurately reflect the actual terrain. The synergistic effect of topography and vegetation at the small-scale mining area was explored based on high-precision data, taking the Shangwan Coal Mine as an example. The high-precision data obtained by UAV support the discovery of the synergistic effect in small-scale mining areas. The fine-scale analysis in this study can clarify the indirect impact of surface subsidence and deformation caused by mining on vegetation. Furthermore, this study can provide scientific guidance for the optimization of ecological restoration measures (especially vegetation restoration measures) according to various terrains in the underground mines.

## 2. Study Area and Datasets

### 2.1. Study Area

**Figure 1:**
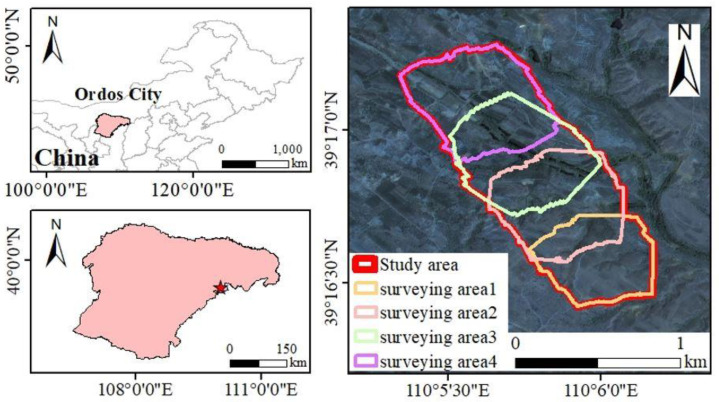
Location of the study area.

The Shangwan Coal Mine is located in Ordos City, Inner Mongolia, China. The mine started to produce coal in 2000, and now it covers an area of 64.21 km^2^. The mine is rich in mineral resources, with geological reserves of 1.23 billion tons and recoverable reserves of 0.83 billion tons. The overall topography in this region is high in the northwest and low in the southeast, with an altitude between 1070 m and 1556 m. The mine is situated in the transitional location of the Mu Us Desert, and it belongs to the semi-arid continental climate in the middle temperate zone. The precipitation is mainly concentrated from July to August, and the annual precipitation is around 400 mm [31]. According to the meteorological station near the mining area, the accumulated precipitation from June to September was 222.72 mm, 322.61 mm, 424.96 mm, 332.98 mm, and 198.35 mm, respectively, in 2017–2020. The vegetation is dominated by perennial grasses and shrubs, including sparse grass, *Hippophae rhamnoides* L., *Ulmus pumila* L., *Thymus mongolicus* Ronn, etc. The mine belongs to the dry denudation zone, and it is mostly aeolian soils. Since 2017, ecological restoration projects (e.g., reconstruction of recreational park and greening of dumping sites) have been carried out on the flat land of the mining area. However, the slope of the mining area is mainly natural vegetation without artificial intervention. The study area consists of four surveying areas in the Shangwan Coal Mine, as shown in Figure 1.

### 2.2. Datasets

**Figure 2:**
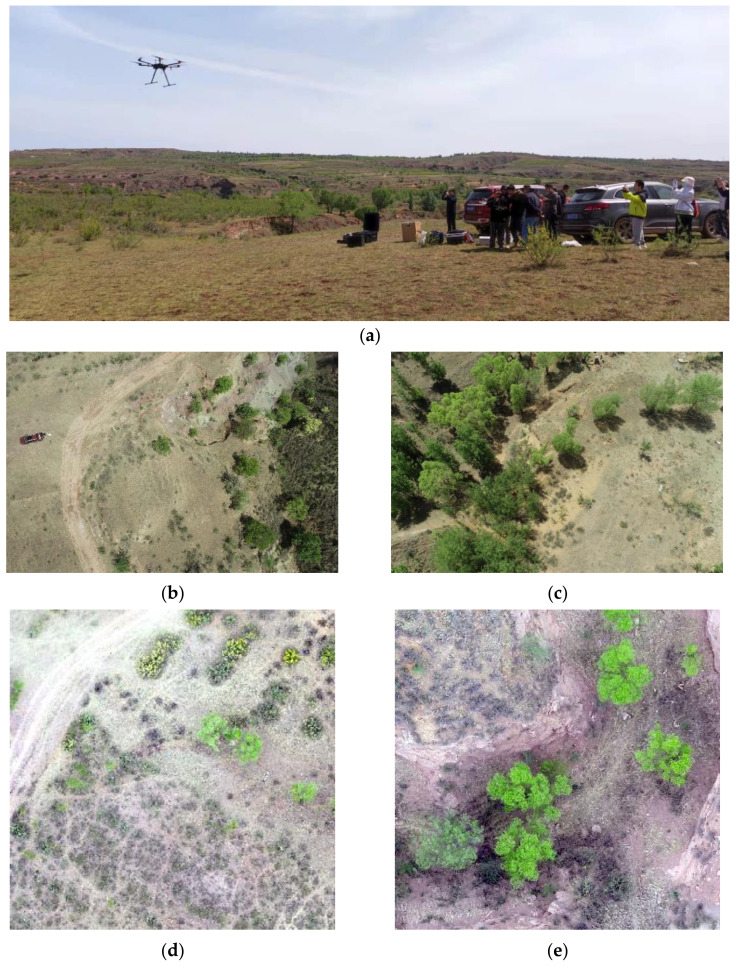
The field photo and the UAV images of the study area: (**a**) field photo; (**b**,**c**) orthophotos; (**d**,**e**) oblique images.

The UAV images and the Landsat remotely sensed images were utilized in this study. The study area was divided into four surveying areas for efficiently taking photos by UAV. The UAV images were collected from 16 May 2021 to 17 May 2021, including orthophotos and oblique images of the four surveying areas, and they were mosaicked and formed a whole UAV image of the study area. The UAV used for aerial photography was the Phantom 4 Pro from SZ DJI Technology Co., Ltd., which has the flight altitude of 100 m and the spatial resolution of 3.45 cm. The field photo, the orthophotos, and the oblique images of the study area are shown in Figure 2. The remotely sensed images include the surface reflectance (SR) dataset of Landsat 8 Operational Land Imager (OLI) in 2017–2021 with a spatial resolution of 30 m.

## 3. Methods

### 3.1. Calculation of NDVI

The NDVI of the study area was calculated online using the Landsat images from 2017 to 2021 on the Google Earth Engine (GEE) [32,33]. The images from June 1 to September 30 in each year were selected to avoid the vegetation change caused by the season difference when the vegetation grows vigorously. The data processing was required for the calculation of the NDVI, including image filtering, cloud removing, and clipping, etc. The “filterDate” method was selected to filter images, and the “CFMask” algorithm was selected for cloud removing, which can be found in the GEE. All images involved in the same year were used to calculate the NDVI, and then the NDVI results of the year were obtained by taking the maximum NDVI at each pixel. The calculation of the NDVI is shown in Equation (1).

(1) $$NDVI=\frac{NIR-R}{NIR+R}$$

where *NIR* is the surface reflectance in the near-infrared band and *R* is the surface reflectance in the red band. The NDVI of the study area was divided into five grades to represent the different vegetation cover [13,34]: −1.0 ≤ NDVI < 0.35 (low-VC), 0.35 ≤ NDVI < 0.48 (slightly low-VC), 0.48 ≤ NDVI < 0.62 (medium-VC), 0.62 ≤ NDVI < 0.75 (slightly high-VC), 0.75 ≤ NDVI < 1.0 (high-VC).

### 3.2. Calculation of Slope and Aspect

The DEM of the study area needed to be generated utilizing UAV images before extracting the slope and aspect [35]. The DEM achieved the digitization of the topography surface utilizing a limited set of 3D spatial coordinates, including three vector factors, X, Y, and Z. X and Y represent the horizontal geographic location of the pixel, and Z represents the elevation of the pixel at the geographic location of (X, Y) [36]. The DEM is to describe the spatial distribution of topography in a particular area, and the topographic factors (i.e., slope, aspect, and slope change rate, etc.) can be extracted from the DEM [37]. The main steps for generating the DEM in this study were as follows: (1) aligning photos, (2) creating a dense point cloud, (3) building grids, (4) constructing textures, and (5) generating DEMs and exporting them. The DEMs of the four surveying areas were acquired and mosaicked to a whole DEM of the study area.

**Figure 3:**
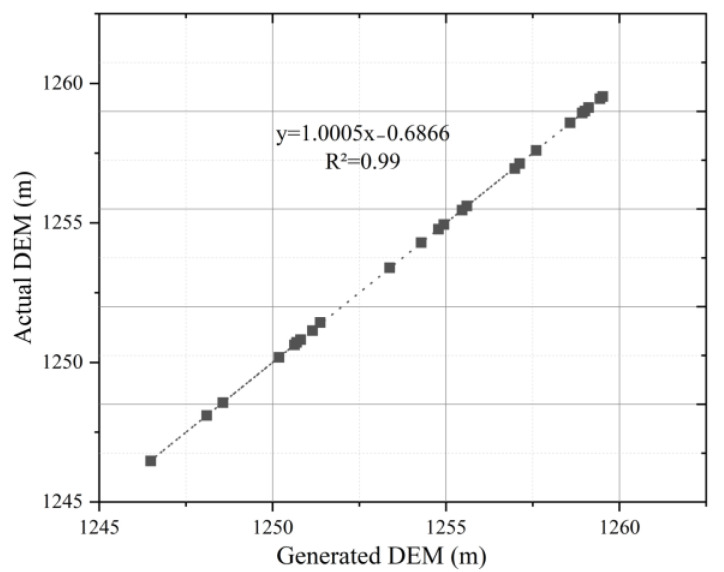
The fitting results of the generated DEM and the actual DEM.

In this paper, the real-time kinematic (RTK) was used to measure the actual evaluation value in the study area for verifying the accuracy of the generated DEMs [38]. The actual value was obtained, and then the generated DEM was fitted with the actual DEM, as shown in Figure 3 (take the surveying area 2 as an example). The goodness-of-fit was 0.99 (R^2^ = 0.99), and the root mean square error (RMSE) was only 0.0171. The data indicated that the deviation between the generated DEM by UAV images and the actual DEM was extremely small. In other words, the generated DEM by UAV had high accuracy and could be used to calculate and analyze the slope and aspect. It also could accurately identify the characteristics of the topography [39].

Slope and aspect are typical topographic factors. Hence, this study selected the two factors to investigate the relationship between topography and vegetation. The slope was calculated using the following Equation (2).

(2) $$\alpha =\arctan\left(\frac{h}{l}\right)$$

where the slope (*α*) is expressed in degrees, and its value is from 0° to 90°. The *h* represents the vertical height of the slope, and *l* represents the horizontal distance of the slope. According to the rules of the International Geographical Union Commission on Geomorphological Surveys and Geomorphological Mapping, the slope was divided into six grades [40]: 0–5° (ground), 5–15° (gentle slope), 15–35° (incline slope), 35–55° (slanted slope), 55–70° (steep slope), and 70–89.9° (rapidly steep slope).

**Figure 4:**
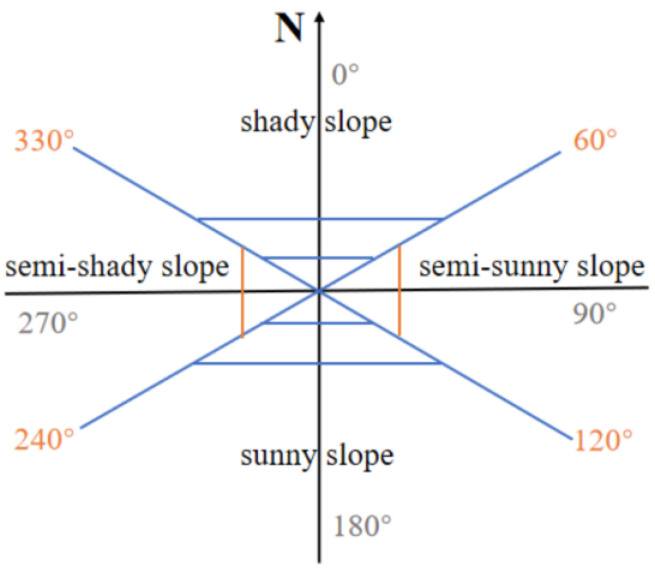
The schematic diagram of four aspect grades.

The range of the aspect was from 0° to 359.9°, where 0°, 90°, 180°, and 270° represent the north, the east, the south, and the west direction, respectively. In addition, the input pixels with zero slope were assigned a −1 aspect, which was labeled “Flat”, and the “Flat” was contained in the “ground” (i.e., slope: 0–5°). The study area lies in the northern hemisphere, and the direct sunlight is from south. Therefore, the south, southeast, and southwest slopes receive the most solar radiation energy. To sum up, the aspect was divided into four grades [41]: 60–120° (semi-sunny slope), 120–240° (sunny slope), 240–330° (semi-shady slope), and 330–60° (shady slope) (Figure 4).

In the study area, 0–5° was classified as the “ground”, and the vegetation changes on the “ground” were analyzed separately. According to the five kinds of slopes (except for the “ground”) and four kinds of aspects, the topography of the study area was divided into 20 combinations, which were “gentle–shady slope”, “gentle–semi-sunny slope”, “gentle–sunny slope”, “gentle–semi-shady slope”, “incline–shady slope”, “incline–semi-sunny slope”, “incline–sunny slope”, “incline–semi-shady slope”, “slanted–shady slope”, “slanted–semi-sunny slope”, “slanted–sunny slope”, “slanted–semi-shady slope”, “steep–shady slope”, “steep–semi-sunny slope”, “steep–sunny slope”, “steep–semi-shady slope”, “rapidly steep–shady slope”, “rapidly steep–semi-sunny slope”, “rapidly steep–sunny slope”, and “rapidly steep–semi-shady slope”, respectively. Therefore, a total of 21 cases (20 combinations + “ground”) were investigated in this paper.

In addition, there was a discrepancy between the spatial resolution of UAV images and the NDVI. Hence, this paper adopted a down-sampling method for harmonizing them: first, the UAV image of the study area was composed of four surveying area images. The four UAV images were resampled to a spatial resolution of 30 m, respectively, and then they were mosaicked into a complete UAV image of the study area. The UAV image and Landsat images in the study area were cropped with the same boundary, and each pixel of the UAV images would correspond to a pixel of the NDVI. Second, the values of the green band and NDVI were counted at the pixel scale, and the relationship between the value of the green band and NDVI was established (Equation (3)). As a result, the correlation coefficient *ρ* was obtained. Third, each pixel of the green band on the original UAV images was multiplied by *ρ*. Finally, the NDVI at a high spatial resolution (i.e., same as UAV images) in the study area was generated.

(3) $$NDVI=\rho \cdot pixel_{green}$$

where NDVI is the NDVI of the pixel, and *pixel_green_* is the value of the green band of UAV image with 30 m resolution. The *ρ* is the correlation coefficient of the value and the NDVI.

## 4. Results

### 4.1. Map of the Vegetation and the Topographic Factors

#### 4.1.1. Spatio-Temporal Distribution of NDVI

**Figure 5:**
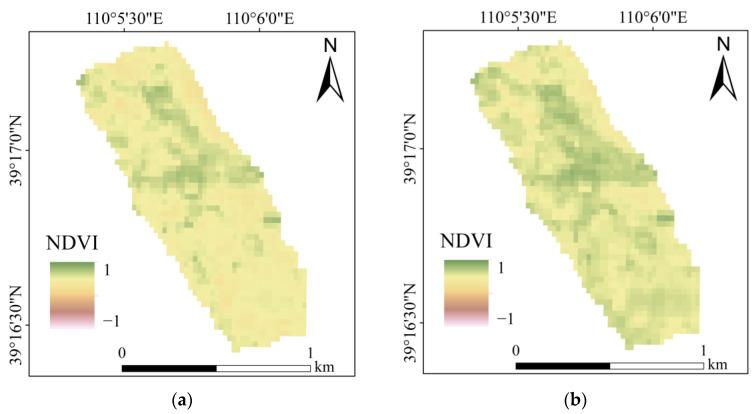

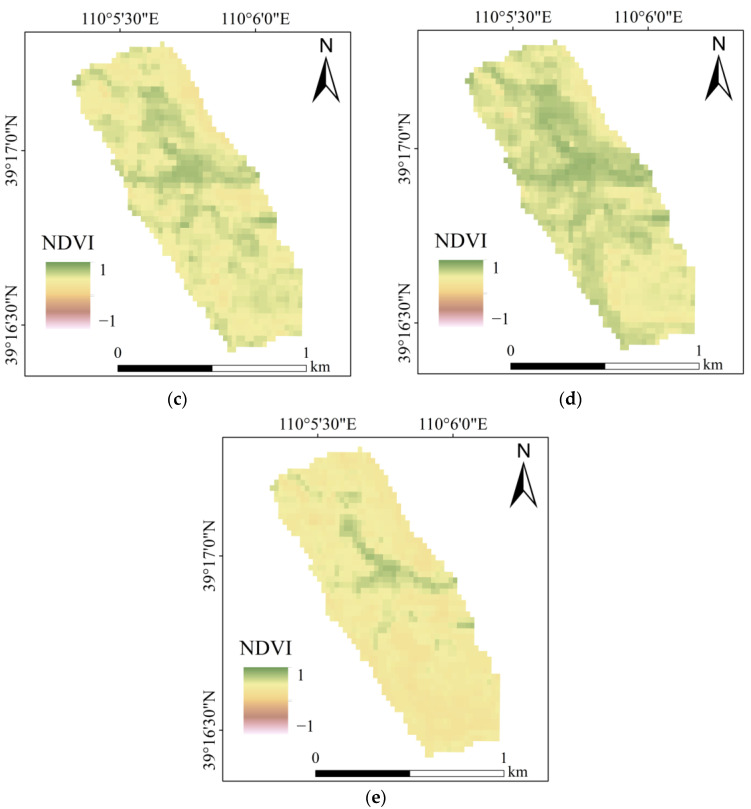
The NDVI of the study area: (**a**) 2017; (**b**) 2018; (**c**) 2019; (**d**) 2020; (**e**) 2021.

**Figure 6:**
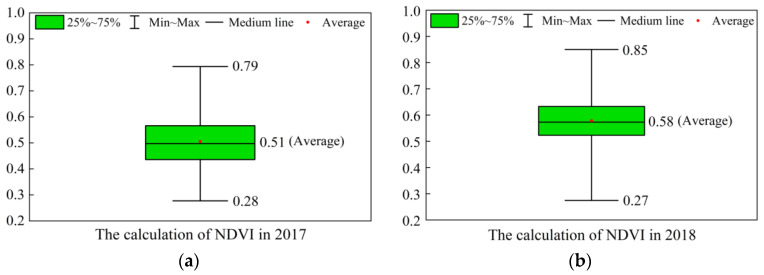

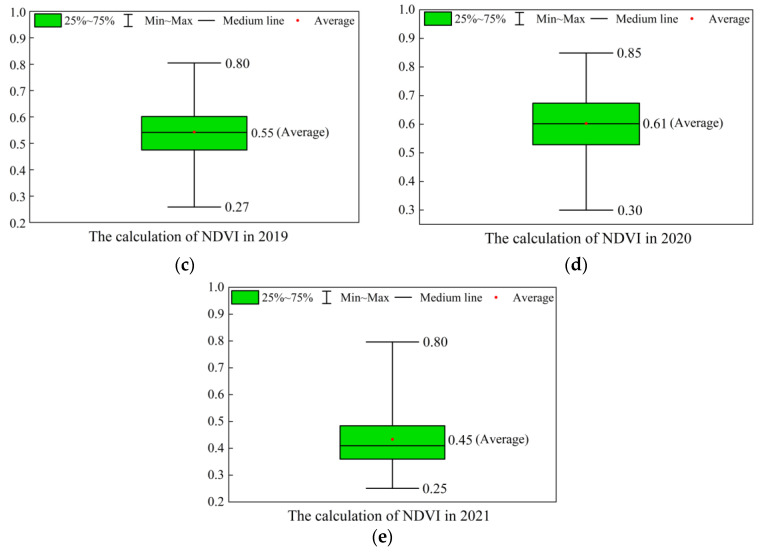
The NDVI distribution in each year: (**a**) 2017; (**b**) 2018; (**c**) 2019; (**d**) 2020; (**e**) 2021.

The results of the NDVI are shown in Figure 5. The statistics of the NDVI in each year are shown in Figure 6. According to the visual effect of Figure 5 and Figure 6, the minimum value of the annual NDVI appeared in 2021, which was 0.25. The maximum value was 0.85, which occurred in 2020 and 2018, respectively. The descending order of the average NDVI from 2017 to 2021 was: 2020 (0.61) > 2018 (0.58) > 2019 (0.55) > 2017 (0.51) > 2021 (0.45).

**Figure 7:**
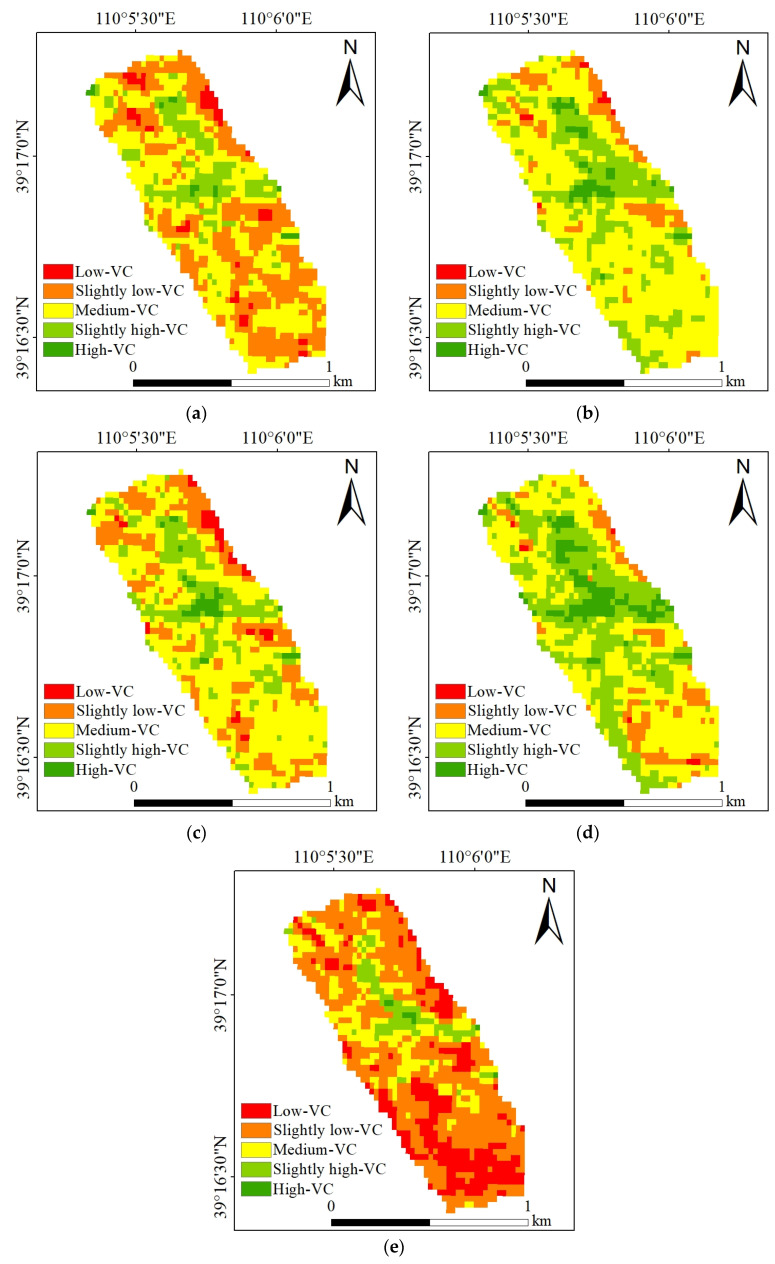
The NDVI grades of the study area: (**a**) 2017; (**b**) 2018; (**c**) 2019; (**d**) 2020; (**e**) 2021.

**Figure 8:**
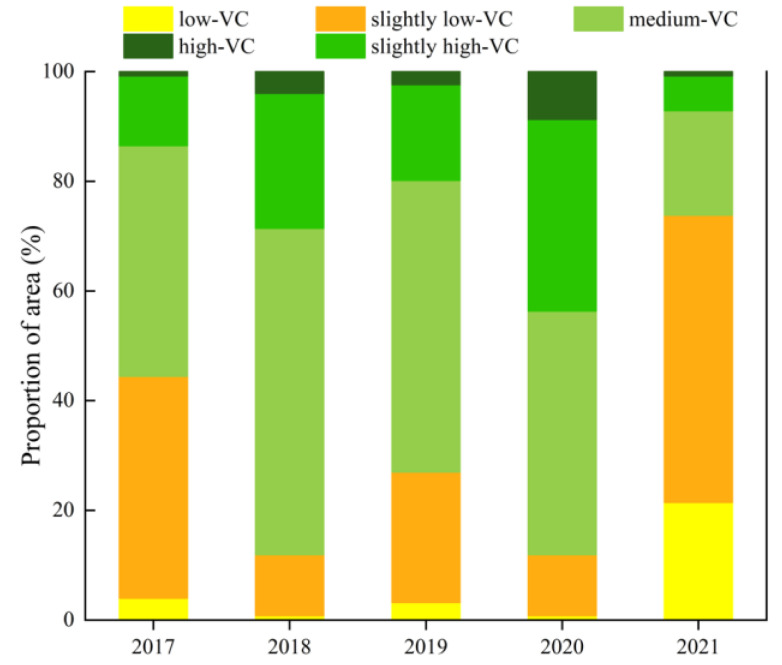
The area proportion of each NDVI grade during 2017–2021.

The grades of the NDVI in the study area are shown in Figure 7. The area proportion of each NDVI grade was counted in each year, as shown in Figure 8. According to the above results, the proportion of “medium-VC” was the largest in 2017–2020, reaching 41.99%, 59.64%, 53.03%, and 44.84%, respectively. However, in 2021, the grade with the largest proportion was “slightly low-VC”, reaching 52.38%, while the proportion of “medium-VC” was only 18.65%. The total proportion of “slightly high-VC” and “high-VC” was more than 10% in 2017–2020, while the total proportion of them was less than 10% in 2021, which was 7.26%.

Overall, the vegetation of the study area has been improving from 2017 to 2020, and the vegetation grew best in 2020. However, in 2021, the vegetation appeared to have serious degradation. The climate change, topographic change, and human activities are the main driving factors of the spatio-temporal variation of the NDVI [42,43]. According to the precipitation of the study area between 2017 and 2021, the accumulated precipitation in the region from June to September between 2017 and 2020 was relatively high. However, the precipitation in 2021 was the lowest, which was only 198.35 mm. Therefore, it is plausible that the serious vegetation degradation in the study area in 2021 was not only related to the intensity of mining activities, but also likely to be strongly related to local climate change.

#### 4.1.2. Map of the Topographic Factors

**Figure 9:**
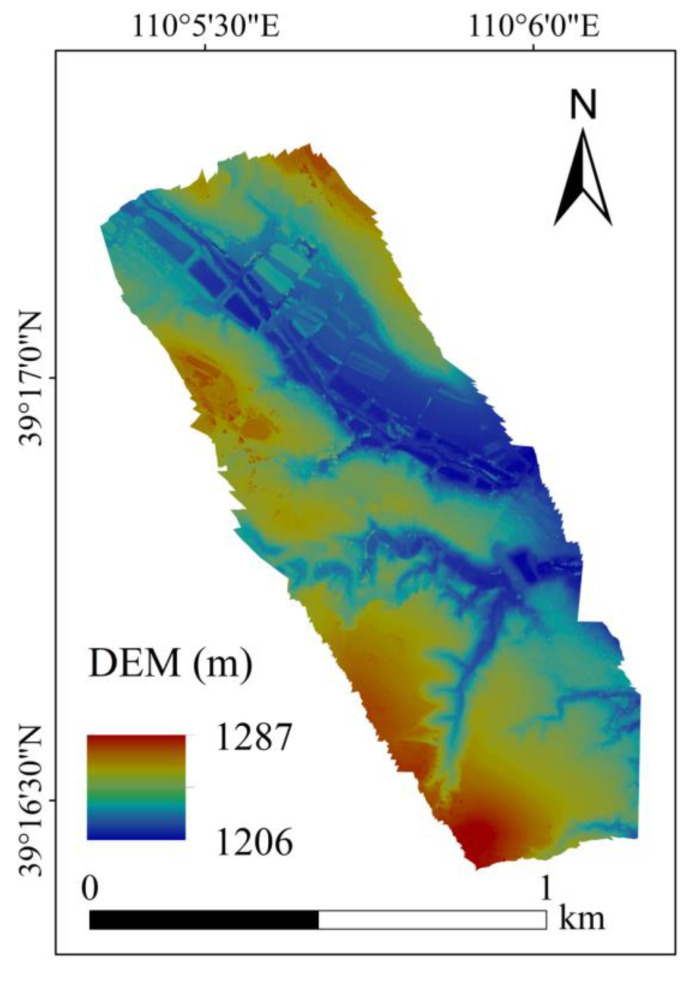
The DEM generated by UAV in the study area.

**Figure 10:**
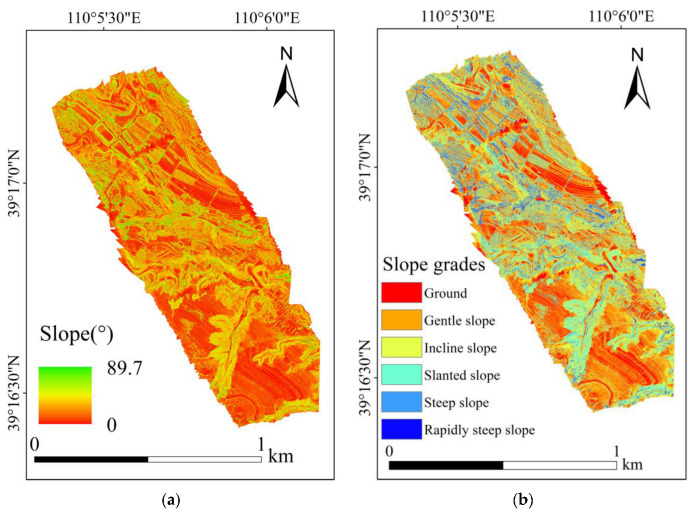
Slope and grades of the study area: (**a**) slope; (**b**) slope grades.

**Figure 11:**
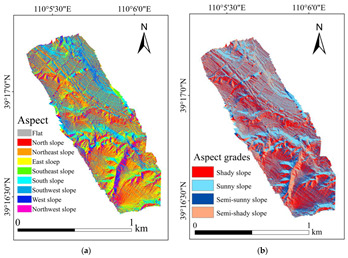
Aspect and grades of the study area: (**a**) aspect; (**b**) aspect grades.

The DEM of the study area generated by UAV is shown in Figure 9. The slope calculated by the generated DEM is shown in Figure 10a, and the graded slope is shown in Figure 10b. The aspect and the grading results are shown in Figure 11.

**Figure 12:**
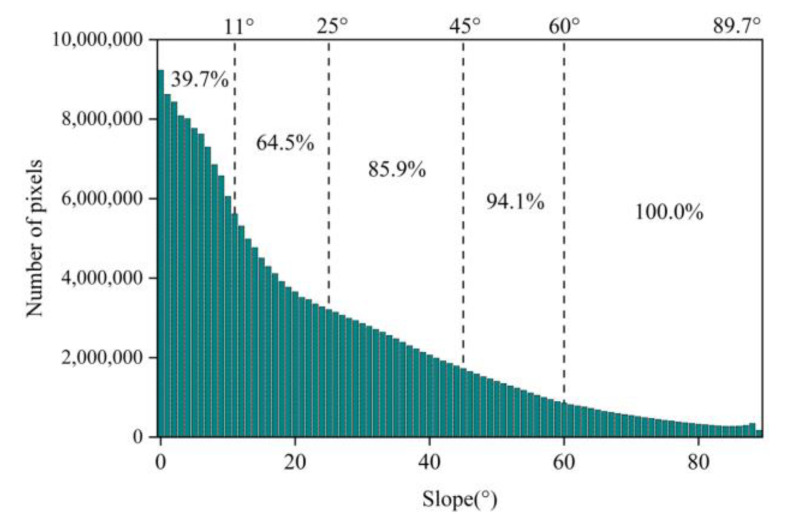
Slope distribution in the study area.

**Figure 13:**
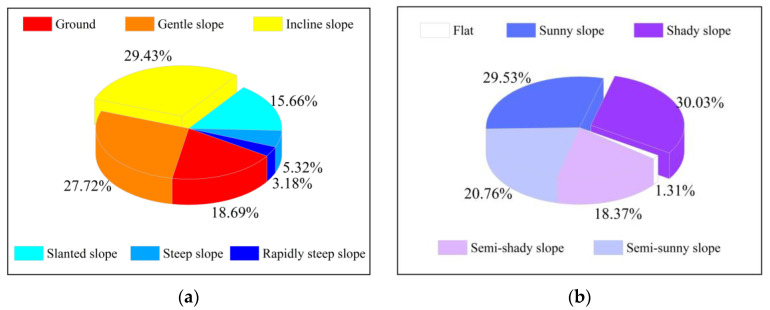
(**a**) Proportion of different slopes; (**b**) proportion of different aspects.

The slope of the study area was counted, as shown in Figure 12. The proportions of pixels for each grade of slope and aspect were counted, as shown in Figure 13.

The elevation of the study area was between 1206 and 1287 m (Figure 9). The slope in the study area ranged from 0 to 89.7°, with a wide fluctuation (Figure 10a). In total, 64% of the slope was below 25°, and about 86% of the slope was below 45° (Figure 12).

In terms of the proportion of each grade for slope and aspect, the slope of 15–35° occupied the largest proportion, and the slope of 70–89° was the least, which was only 3.18%. More than 75% of the slopes were between 0 and 35°. The proportion of “shady slope” was the highest, which was 30.03% (Figure 13a). In addition, the proportion of “sunny slope” was very similar to the “shady slope”, which was 29.53%. Comparatively speaking, the “semi-sunny slope” and “semi-shady slope” were relatively less, accounting for about 20%, respectively (Figure 13b).

Although there were almost vertical slopes in the study area, they were very rare, and most slopes were relatively gentle.

**Figure 14:**
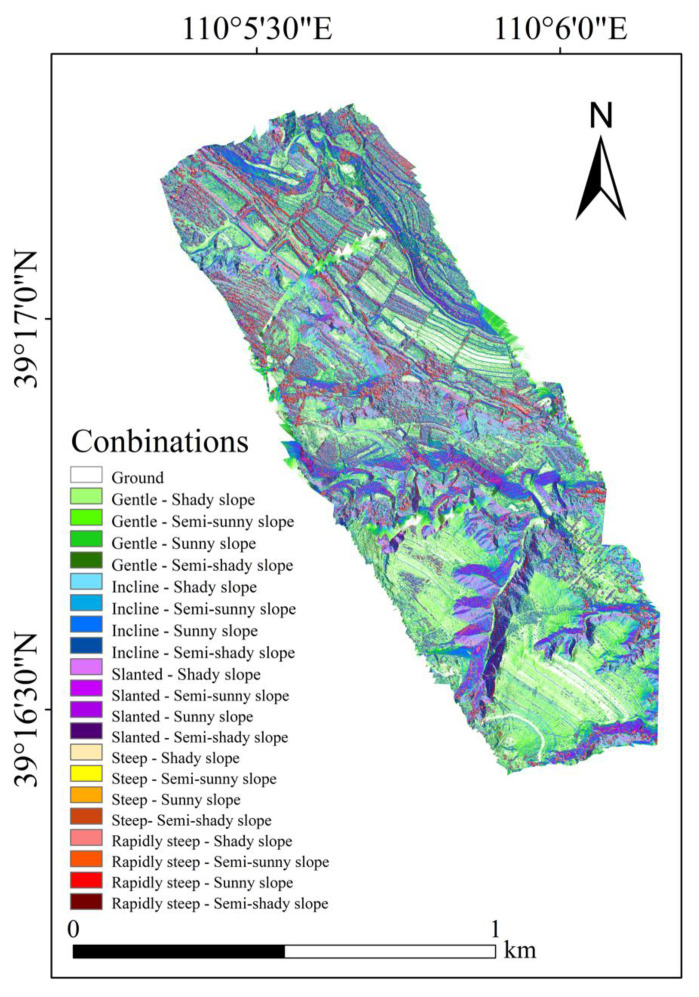
The 20 combinations of slope and aspect.

**Figure 15:**
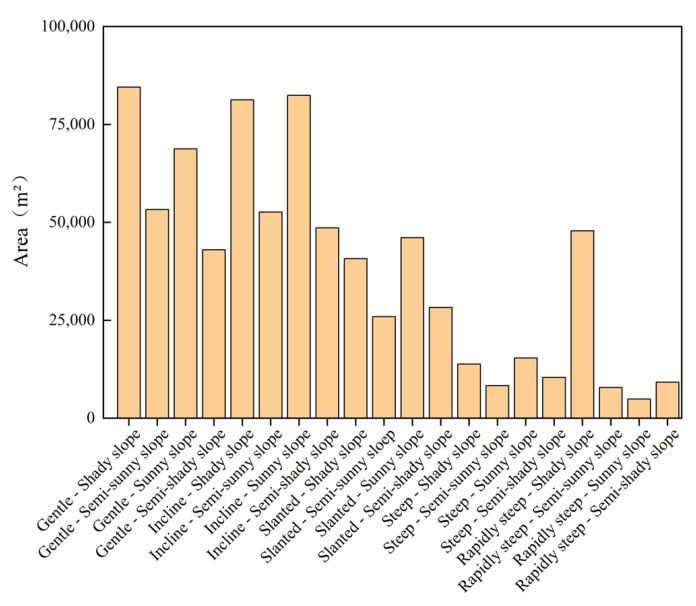
Area of 20 combinations.

Figure 14 shows the 20 types of combinations between the slope and aspect. Figure 15 shows the area statistics of the different combinations. The area of the combinations with “gentle slope”, “incline slope”, and “slanted slope” was generally large (Figure 15). For example, the area of “gentle–shady slope”, “incline–shady slope”, and “incline–sunny slope” was significantly larger than the other combinations, which were 84,498 m^2^, 81,259 m^2^, and 82,417 m^2^, respectively. However, the area of the combination with “steep slope” and “rapidly steep slope” was very small, among which the area of the “rapidly steep–sunny slope” was the smallest, only 4893 m^2^.

### 4.2. Influence of Slope on Vegetation

**Figure 16:**
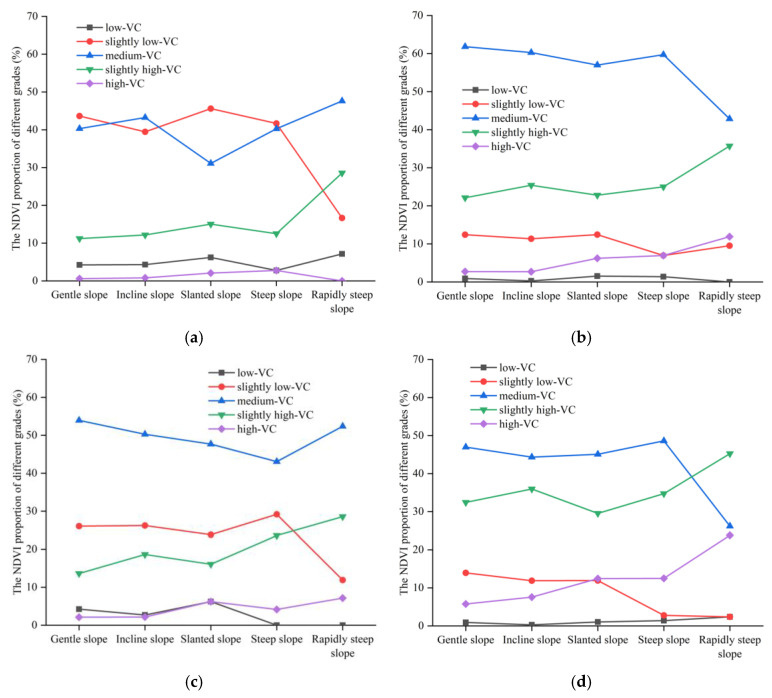

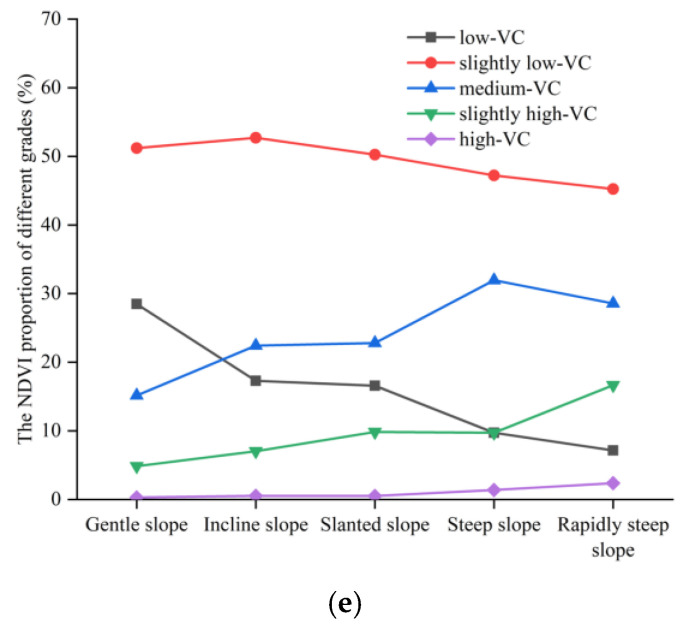
The proportion of different vegetation cover grades on different slopes: (**a**) 2017; (**b**) 2018; (**c**) 2019; (**d**) 2020; (**e**) 2021.

The proportion of different vegetation cover grades on different slopes in 2017–2021 is shown in Figure 16. The total proportion of “slightly low-VC”, “medium-VC”, and “slightly high-VC” in each slope reached 90% in 2017. With the increase in slope, the proportion of “slightly low-VC” showed a decreasing trend. On the contrary, the proportion of “medium-VC” and “slightly high-VC” showed an increasing trend. In 2018, all slopes were dominated by “medium-VC” and “slightly high-VC”, and the sum of them accounted for about 80% in each slope. With the increase in slope, the proportion of “low-VC” and “slightly low-VC” changed slightly. The proportion of “medium-VC” showed a decreasing trend, and the proportion of “slightly high-VC” and “high-VC” showed an increasing trend. The main grades were “slightly low-VC”, “medium-VC”, and “slightly high-VC” in 2019, and the sum of the proportion was about 90%. By 2020, the “gentle slope”, “incline slope”, and “steep slope” were dominated by “slightly low-VC”, “medium-VC”, and “slightly high-VC”. The “steep slope” and “rapidly steep slope” were dominated by “medium-VC”, “slightly high-VC”, and “high-VC”. The proportions of “slightly low-VC” were the largest in 2021 in each slope, which were 51.2%, 52.7%, 50.3%, 47.2%, and 45.2%, respectively. With the increase in slope, the proportion of “low-VC” and “slightly low-VC” showed a decreasing trend. The proportion of “medium-VC”, “slightly high-VC”, and “high-VC” showed an increasing trend.

Overall, the NDVI of each slope grade in the study area was dominated by “slightly low-VC”, “medium-VC”, and “slightly high-VC” in the past five years. With the increasing of slope, the proportion of “slightly low-VC” showed a decreasing trend. However, the proportion of “slightly high-VC” showed an increasing trend.

**Figure 17:**
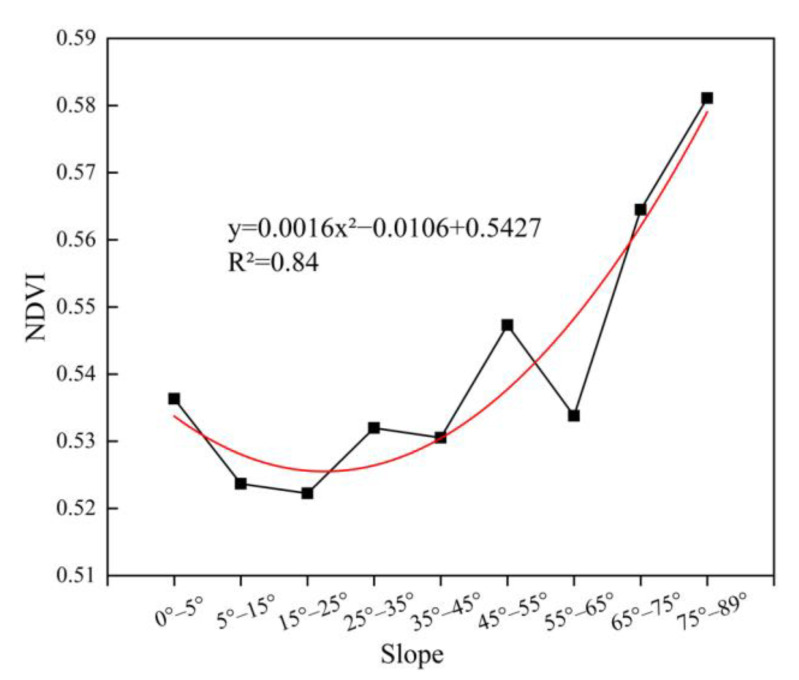
The relationship of the slope and NDVI.

The average NDVI of different slopes was counted, as shown in Figure 17. In the study area, the slope was positively correlated with the NDVI overall as the slope was more than 5°. The higher the slope, the larger the NDVI. The NDVI change was found to be positively affected by human activities, and sometimes human activities had a negative impact on the change of the NDVI [20,22,44]. In this study area, there were fewer human activities (i.e., surface plant, transportation channel, etc.) on steep slopes than those on relatively gentle slopes under the same climatic conditions. Therefore, the NDVI grew better on the steeper slopes.

### 4.3. Influence of Aspect on Vegetation

**Figure 18:**
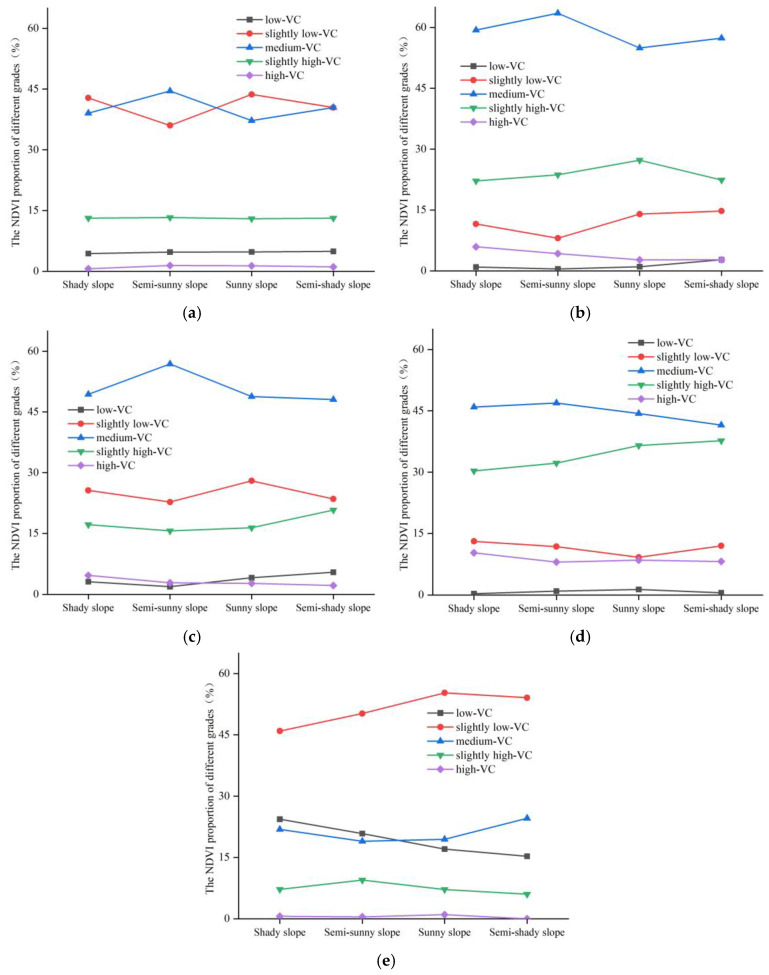
The proportion of different vegetation cover grades on different aspects: (**a**) 2017; (**b**) 2018; (**c**) 2019; (**d**) 2020; (**e**) 2021.

The proportions of different vegetation cover grades on different aspects in 2017–2021 are shown in Figure 18. In 2017, “slightly low-VC” and “medium-VC” were dominant vegetation cover grades in each aspect, and the sum of the proportion for the two grades exceeded 80% in each aspect. The curves of “slightly low-VC” and “medium-VC” fluctuated slightly, but the curves of “low-VC”, “slightly high-VC”, and “high-VC” were basically stable in each aspect. All the aspects were dominated by “medium-VC” in 2018 and 2019, with a proportion of about 60% and 50%, respectively. By 2020, the NDVI grades of different aspects were mainly “medium-VC” and “slightly high-VC”, and the sum of the proportion was 80%. However, the main NDVI grade of different aspects was “slightly low-VC” in 2021, which accounted for about 50%. In this year, the proportion of “high-VC” was almost 0.

In general, the grades of different aspects in the study area were mainly “medium-VC” in 2017–2020, and “slightly low-VC” in 2021. Furthermore, “sunny slope” or “semi-sunny slope” had a higher NDVI ratio. It may be because the “sunny slope” and “semi-sunny slope” received more sunshine than the “shady slope” and “semi-shady slope”, and the vegetation obtained a more favorable growing environment. Therefore, the vegetation grew more luxuriantly.

### 4.4. The NDVI Cover under 21 Types of Combinations

#### 4.4.1. Vegetation Cover on “Ground”

In this paper, 0–5° was divided into the “ground”. Due to the low slope, the aspect factor was not considered and in the combinations of “ground”, different aspects were not involved. Therefore, the vegetation on the “ground” was analyzed separately.

**Figure 19:**
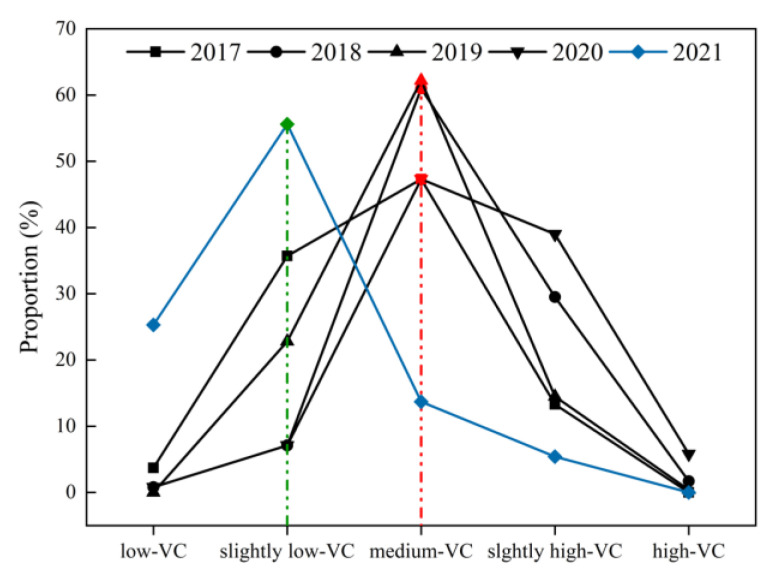
The proportion of different vegetation cover grades on “ground” from 2017 to 2021.

Figure 19 shows the proportion of different vegetation cover grades on the “ground” from 2017 to 2021. The vegetation cover grade on the “ground” from 2017 to 2020 was mainly “medium-VC”. Especially in 2018 and 2019, the “medium-VC” was up to more than 60%. In addition, the area of “low-VC” in these two years was almost 0 on the “ground”. By 2021, the study area was dominated by “low-VC” on the “ground”. The total proportion of “low-VC” and “slightly low-VC” reached 80%. The NDVI on the “ground” was the most between 0.48 and 0.62, and the vegetation growth was normal.

#### 4.4.2. Vegetation Cover under 20 Combinations

**Figure 20:**
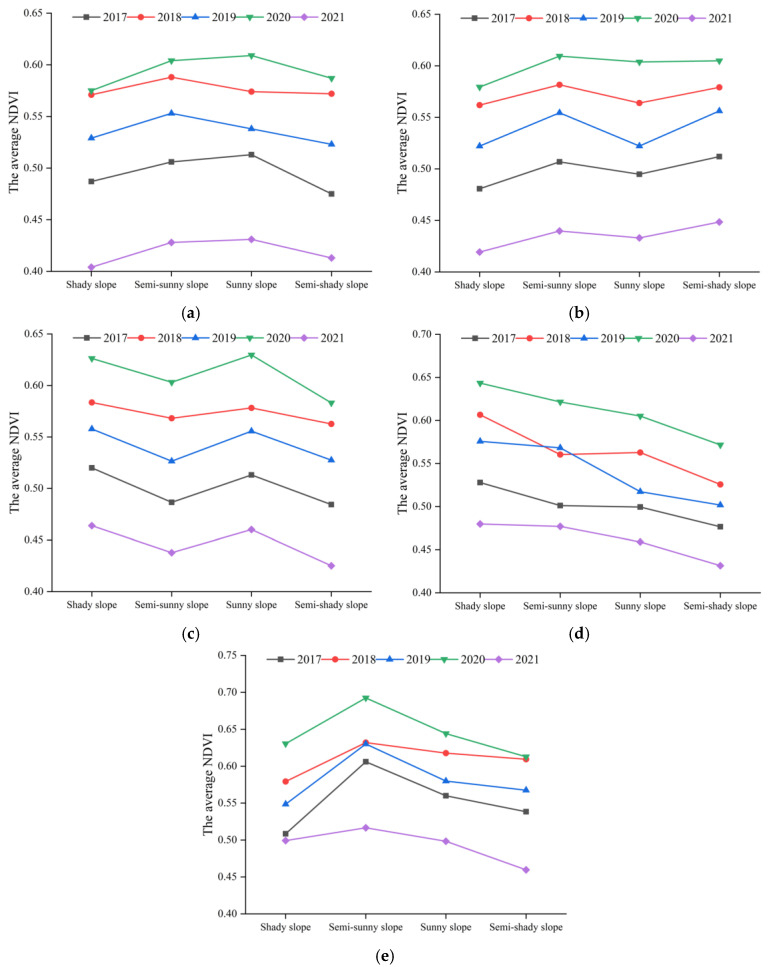
NDVI of different slopes and aspects: (**a**) gentle slope—different aspects; (**b**) incline slope—different aspects; (**c**) slanted slope—different aspects; (**d**) steep slope—different aspects; (**e**) rapidly steep slope—different aspects.

For the average NDVI of the gentle slope, different aspects from 2017 to 2021 were calculated and counted, as shown in Figure 20a. In general, the changing trend of the average NDVI was not obvious. The NDVI exhibited higher values on the “semi-sunny slope” and the “sunny slope”.

For the average NDVI of the incline slope, different aspects from 2017 to 2021 are shown in Figure 20b. The NDVI generally showed a steady increasing trend in each year. The NDVI was better on the “semi-sunny slope”, where the NDVI in 2017–2020 was 0.51, 0.58, 0.55, 0.61, and 0.44, respectively.

Figure 20c shows the average NDVI of the slanted slope with different aspects from 2017 to 2021. All five curves showed a fluctuating decreasing trend, but the “sunny slope” had a better NDVI at the same time.

Figure 20d shows the NDVI of a steep slope with different aspects from 2017 to 2021. The curves in the figure show an obvious decreasing trend. The maximum NDVI appeared in the “shady slope”, and it was followed by the “semi-sunny slope”.

The average NDVI of the rapidly steep slope with different aspects from 2017 to 2021 is shown in Figure 20e. It visually reflects that the maximum NDVI occurred on the “semi-sunny slope”, where the NDVI in 2017–2020 was 0.61, 0.63, 0.63, 0.69, and 0.52, respectively.

In Figure 20, the vertical fluctuation of each curve in (a), (b), and (c) was smaller than those in (d) (e). The aspects had less influence on vegetation growth in the study area when the types were “gentle slope”, “incline slope”, and “slanted slope”. When the slopes were “steep slope” and “rapidly steep slope”, the aspect had an obvious influence on vegetation growth.

**Figure 21:**
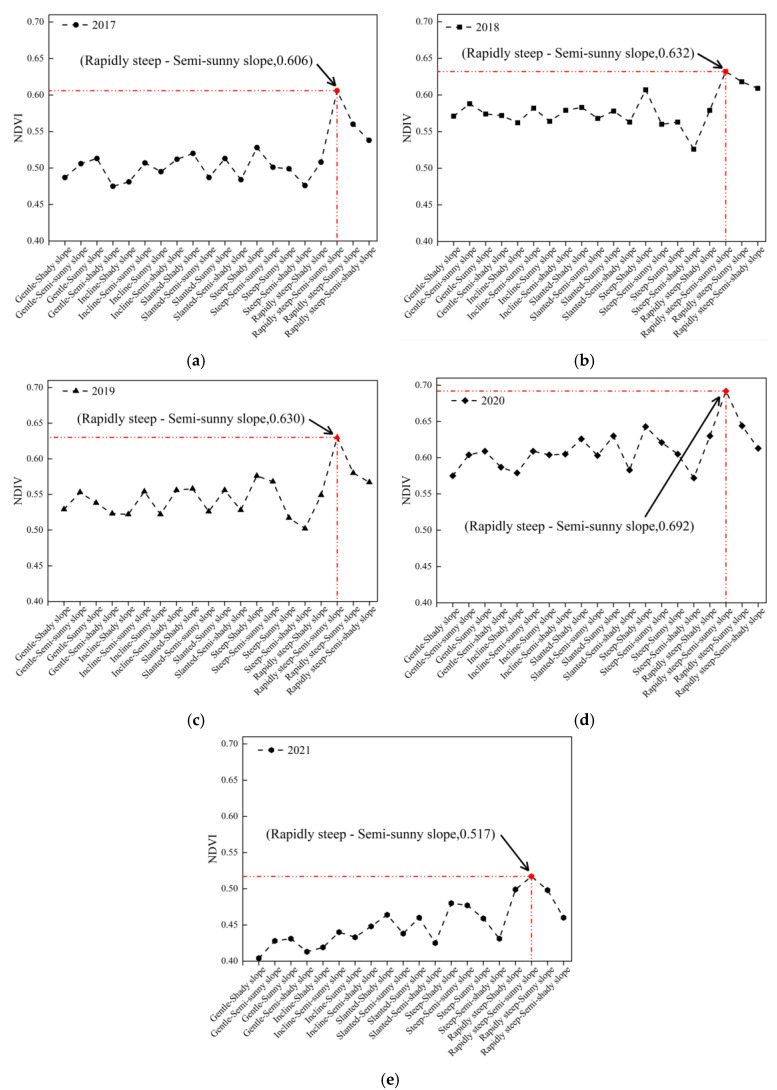
NDVI of 20 combinations in different years: (**a**) 2017; (**b**) 2018; (**c**) 2019; (**d**) 2020; (**e**) 2021.

The NDVI curves were plotted in each year under 20 combinations in the study area to understand which combination of topographic factors had the best vegetation growth, as shown in Figure 21. Compared to the overall situation of the five-year curves, the curve in 2020 was the furthest away from the *X*-axis, and the curve in 2021 was the closest to the *X*-axis. In 2017–2021, the combination of “rapidly steep–semi-sunny slope” had the highest NDVI in each year, and the order was as follows: 2020 (0.692) > 2018 (0.632) > 2019 (0.630) > 2017 (0.606) > 2021 (0.517).

## 5. Discussion

This paper makes up for some shortcomings in existing publications, and the contributions of this paper are discussed as follows.

(1) Acquisition of high-precision topographic data. The UAV was used to obtain the high-precision DEM, slope, and aspect of the study area. The accuracy of the DEM generated by the UAV was verified to be credible, proving that the DEM generated using the UAV is highly consistent with the actual elevation, and it can be used to conduct relevant studies.

(2) Mine-scale synergistic effect analysis based on high-precision data. The synergistic effect between the topographic factors and vegetation index in the underground coal mine area was explored utilizing high-precision data, which provided a technical support for more subsequent studies, and a new solution to the current situation that the accuracy of topographic factors used in existing studies was not usually sufficient to support the research objectives. Furthermore, it can also provide a decision basis for local restoration projects.

There existed a positive correlation between the slope and NDVI when the slope was more than 5° (Figure 17). The positive correlation is consistent with the study of the natural mountain area by Zhu et al. However, the NDVI showed a slightly decreasing trend and then increasing trend with the increase in slope in the study area. Zhu et al. showed that the NDVI first increased and then slightly decreased along with the slope. It indicated that the NDVI in this study area was more sensitive at higher slopes, while it was more sensitive at a gentle slope in the Qinling Mountains. The reason for this difference is probably related to the subsidence of the underground coal mine and the territory where the study area is located. Therefore, it is necessary to explore the relationship between vegetation and topography in the underground mining area, which has an important referential value for adjusting measures of ecological restoration according to the local resources and environment in the mining area. In this paper, the regions with low slope had less vegetation cover, because sandy land was predominated in the low-slope regions by field investigation. The environment of the soil was not suitable for vegetation growth. In addition, these regions had intensive human activities, resulting in more negative impacts on vegetation growth. As a result, it led to the poorer vegetation cover on the gentle slope of the study area.

The combinations with the “sunny slope” and “semi-sunny slope” had higher vegetation cover (Figure 20). The reason is that the “sunny slope” and “semi-sunny slope” received sufficient sunlight, which created an advantageous environment for the vegetation growth. This is consistent with the findings of Zhou et al. who considered that variation in vegetation cover on different aspects was caused by differences in the distribution of solar radiation, precipitation, and human activities [45]. However, the results of the above publications were obtained in urban or mountainous areas, not mining areas. One of the contributions of this paper is to realize and confirm the similar conclusions in the underground mining area with subsidence. In addition, the combination of “rapidly steep–semi-sunny slope” had the highest vegetation cover in 2017–2021 (Figure 21). This is because the “sunny slope” received more direct sunlight than the “semi-sunny slope”, so that the evaporation of water in soil on the “sunny slope” was greater than that on the “semi-sunny slope”. Therefore, the “semi-sunny slope” can preferably maintain the relationship between sunlight and water, and make vegetation grow better.

However, there are still some limitations that need further study. The NDVI was acquired in GEE from 2017 to 2021, and the photos used to generate the DEM were taken by UAV from May 16, 2021 to May 17, 2021. This study assumed that the DEM within the study area was essentially constant in the analysis due to the small scope of the study area and the fact that the elevation changes were not significant within this five-year period. Subsequent studies can collect multiple years of high-precision DEM data to match the NDVI. In addition, the long time series of data was also needed to meet the analytical needs of the mine with a long mining history.

## 6. Conclusions

The high-precision DEM with a spatial resolution of 3.45 cm in the study area was obtained using the UAV images, and the slope and aspect were further calculated by the DEM. The slope and aspect of the study area were divided into 20 types of combinations. Subsequently, the calculated NDVI was down-sampled for obtaining data with the same spatial resolution as the topographic factors. Finally, the relationships between topography and vegetation were analyzed in the Shangwan Underground Coal Mine. The following conclusions were acquired.

(1) The “slightly low-VC”, “medium-VC”, and “slightly high-VC” were the main vegetation cover types in the study area from 2017 to 2021. There was a positive correlation relationship between the slope and NDVI when the slope was more than 5°.

(2) In the case of different slopes, the “sunny slope” and “semi-sunny slope” were more suitable for vegetation growth. The effect of the aspect on vegetation was smaller at low slopes and greater at higher slopes.

(3) Among the 20 combinations of the slope and aspect, the “rapidly steep–semi-sunny slope” was the most favorable for vegetation growth in the study area.

Based on the high-resolution topographic data, this paper discovered the relationship between topography and the NDVI in the Shangwan Coal Mine and revealed the influence of slope and aspect on vegetation growth. The conclusions are conducive to optimizing the selection of vegetation planting modes and improving the ecological environment in the underground coal mine. In the future, more years of topographic data will be used to further explore the quantitative relationship between topographic factors and vegetation index in the underground coal mine.

## Data Availability

Data are available from the corresponding author upon reasonable request.
